# Innate immune functions of microglia isolated from human glioma patients

**DOI:** 10.1186/1479-5876-4-15

**Published:** 2006-03-30

**Authors:** S Farzana Hussain, David Yang, Dima Suki, Elizabeth Grimm, Amy B Heimberger

**Affiliations:** 1 Department of Neurosurgery – Unit 442, The University of Texas M.D. Anderson Cancer Center, 1515 Holcombe Blvd, Houston, TX 77030, USA; 2 Department of Experimental Therapeutics, The University of Texas M.D. Anderson Cancer Center, 1515 Holcombe Blvd, Houston, TX 77030, USA

## Abstract

**Background:**

Innate immunity is considered the first line of host defense and microglia presumably play a critical role in mediating potent innate immune responses to traumatic and infectious challenges in the human brain. Fundamental impairments of the adaptive immune system in glioma patients have been investigated; however, it is unknown whether microglia are capable of innate immunity and subsequent adaptive anti-tumor immune responses within the immunosuppressive tumor micro-environment of human glioma patients. We therefore undertook a novel characterization of the innate immune phenotype and function of freshly isolated human glioma-infiltrating microglia (GIM).

**Methods:**

GIM were isolated by sequential Percoll purification from patient tumors immediately after surgical resection. Flow cytometry, phagocytosis and tumor cytotoxicity assays were used to analyze the phenotype and function of these cells.

**Results:**

GIM expressed significant levels of Toll-like receptors (TLRs), however they do not secrete any of the cytokines (IL-1β, IL-6, TNF-α) critical in developing effective innate immune responses. Similar to innate macrophage functions, GIM can mediate phagocytosis and non-MHC restricted cytotoxicity. However, they were statistically less able to mediate tumor cytotoxicity compared to microglia isolated from normal brain. In addition, the expression of Fas ligand (FasL) was low to absent, indicating that apoptosis of the incoming lymphocyte population may not be a predominant mode of immunosuppression by microglia.

**Conclusion:**

We show for the first time that despite the immunosuppressive environment of human gliomas, GIM are capable of innate immune responses such as phagocytosis, cytotoxicity and TLR expression but yet are not competent in secreting key cytokines. Further understanding of these innate immune functions could play a critical role in understanding and developing effective immunotherapies to malignant human gliomas.

## Background

Malignant gliomas are the most common type of primary brain tumors and glioblastoma multiforme accounts for more than 50% of all intracranial gliomas [1]. These tumors are extremely aggressive and are characterized by diffuse infiltration of the brain parenchyma, recurrent growth, and an extremely poor prognosis for survival. Although glioblastoma multiforme is immunogenic [2-4], immune-mediated eradication does not occur, and attempts at immunotherapy directed against brain tumors have been minimally successful thus far [5-7]. Previously characterized impairments in glioma immunity have included low peripheral lymphocyte counts, reduced delayed type hypersensitivity reactions to recall antigens, impaired mitogen-induced blastogenic responses by peripheral blood mononuclear cells (PBMCs), and increased CD8^+ ^suppressor T cells [8]. Adaptive immune responses are noticeably deficient, with diminished responsiveness of peripheral T cells associated with impaired early transmembrane signaling through the T-cell receptor/CD3 complex [9]. In addition, diminished induction of immunoglobulin synthesis by B cells *in vitro *from the peripheral blood of patients with intracranial tumors appears to be related to diminished T-helper activity [10]. Although immune impairments have been identified within the adaptive arm, few studies have examined for potential deficits in the innate arm, especially within the context of immunosuppressed glioma patients.

Innate immunity is the initial antigen-nonspecific response that results in the rapid production of effector cytokines and is one of the prerequisites for triggering effective adaptive antitumor immune responses. Soluble factors produced by gliomas, such as immunosuppressive cytokines (e.g., transforming growth factor [TGF-β ] and interleukin [IL-10]), presumably impair other cells participating in innate immunologic responses. Microglia are the most prominent immune cell within the CNS, however, it is not known whether microglia, within the immunosuppressive tumor environment, are capable of activated or functional innate immune responses. Microglia are unique to the central nervous system (CNS) and account for as much as 20% of the non-neuronal cell population [11]. Microglia are defined as being CD11b/c^+^CD45^low^, macrophages as CD11b/c^+^CD45^high^, and lymphocytes as CD11b/c^-^CD45^high^[12]. There is not a distinct universally accepted histological marker to distinguish macrophages from microglia. However, macrophages are believed to be activated microglia within the CNS. Rodent studies have shown that microglia play a critical effector role in rapidly responding to certain autoimmune and viral infections [13]. Microglia are thought to be capable of phagocytosis, cytotoxicity, and other pro-inflammatory innate effector functions [14]. The cells of the innate immune system, especially macrophages, use Toll-like receptors (TLRs) to recognize microbial or non-self factors, such as pathogen-associated molecular patterns. TLRs are not constitutively expressed in the brain parenchyma [15], but cultured primary murine microglia cells express mRNA encoding TLRs, which are strongly activated after stimulation with their specific agonists (lipopolysaccharide (LPS) (TLR-4), peptidoglycan (TLR-2), dsRNA (TLR-3), or CpG motifs (TLR-9)) [16]. Signaling through these receptors is coupled to gene transcription processes and has powerful immunomodulatory effects that can result in the activation of anti-tumor immune responses. Murine microglia have been shown to express the mRNA for all TLRs (TLR1-9) [13]. However, these microglia cell lines have been propagated extensively in culture and thus their phenotype and function could be markedly altered from the characteristics that they would normally have *in situ*.

The soluble expression of FasL fails to induce inflammatory responses. In contrast, when FasL is expressed on the surface of tumor cells, neutrophil-mediated inflammation is triggered initiating a vigorous innate immune response [17,18]. The complex immunological role of FasL has been confounded by a recent report that tumor expression of FasL impairs NK activation [19]. Expression of FasL has been shown to maintain immune privilege though inducing apoptosis of infiltrating Fas positive effector T cells. In the murine G26 model system, 50% of the total FasL-positive expression in intracranial tumors was accounted for by microglia, and neutralization of FasL resulted in a significant increase in the number of tumor-infiltrating lymphocytes [20]. This would indicate that microglia may play a role in down regulating innate immune responses within the CNS.

In this study, for the first time, we have characterized the innate immune phenotype and function of microglia isolated from human glioma tissue immediately after surgical resection. Using sequential Percoll density gradients, the purity of GIM was determined on the basis of previously established CD11b^+^CD45^low ^parameters [21]. The ability of GIM to participate in innate immune function was assessed by analyzing the surface expression of TLRs, their ability to mediate tumor cytotoxicity, phagocytosis and expression of FasL. Similar to findings in the murine systems, GIM did express TLR, however GIM did not elaborate cytokines reflective of innate activation. In marked contrast to findings within the murine system, GIM do not express FasL. Furthermore, although GIM could mediate non-MHC restricted cytotoxicity, microglia isolated from normal brain were statistically more efficient at tumor cytotoxicity. By characterizing the activation state and functioning of GIM, we can not only comprehensively identify their role in immune responses to human gliomas, but we can also begin to develop ways in which they can be manipulated to successfully design and optimize immunotherapeutic strategies against malignant brain tumors.

## Methods

### Human subjects

Patients' tumors (n = 50) were graded pathologically as glioblastoma multiforme by a neuropathologist according to the WHO classification [22,23]. Fresh normal brain tissue (n = 6) can only be rarely obtained since for most patients efforts are made by the neurosurgeon to preserve this tissue. On occasion, normal brain tissue was obtained during an approach to a benign tumor such as a sphenoid wing meningioma, a well-circumscribed metastasis or a low-grade lesion such as an oligodendroglioma. Additionally, peritumoral GBM tissue was also obtained and separately analyzed, however because this tissue could harbor microscopic tumor it was not characterized as normal for the purposes of this study. The resected normal tissue demonstrated no evidence of increased signal on flair MRI pre-operative imaging. The brain architecture was inspected microscopically intraoperatively by an experienced neurosurgeon (A.B.H.) and found to be consistent with normal brain tissue prior to submission to the laboratory. Peripheral blood was drawn from the patients intraoperatively. This study was conducted under protocol # LAB03-0687, which was approved by the institutional review board of The University of Texas M.D. Anderson Cancer Center, and informed consent was obtained.

### Prioritization of characterization

Microglia could never be isolated in sufficient numbers from a single GBM specimen to perform all of the assays concurrently. Furthermore, there is significant variability in the numbers of microglia obtained from each clinical specimen that could not be predicted preoperatively. A minimum of 5 grams of tissue was necessary in order to isolate sufficient microglia for the basic phenotypic characterizations. Purity was established on all specimens regardless of the assay priority. Fresh surgical tissues were analyzed as follows: 1) If a normal and GBM specimen could be obtained simultaneously then priority went to performing the cytotoxicity assays. In two years, this scenario was very rare even at a hospital specializing in neuro-oncological neurosurgery. Routinely drawn intraoperative peripheral blood was accessed for the controls. 2) For specimens > 5 grams, the microglia were phenotypically and functionally characterized.

### Isolation of microglia from human brain tumor tissue samples

Microglia were purified using modifications to an isolation technique previously described [21] after evaluation of the phenotype of each interphase. This technique minimized artificial activation of the microglia and these cells were isolated usually within three hours of surgical resection. Briefly, after surgical resection, freshly isolated tumor, peritumoral, or normal brain tissue was mechanically dissociated through a stainless steel sieve. The dissociated material was centrifuged and the pellet was washed. Cells were layered onto an isotonic Percoll (Amersham Biosciences, Uppsala, Sweden) gradient diluted to 1.095 g/mL and overlaid with 1.03 g/mL Percoll. After centrifugation, the visible cell layer between the 1.095 g/mL and 1.03 g/mL layers was removed, washed, layered on top of a second gradient (2-mL steps of isotonic Percoll diluted to 1.12, 1.088, 1.072, 1.065, and 1.03 g/mL densities) and centrifuged. Microglia were collected from the interface between the 1.065 g/mL and 1.03 g/mL layers and washed. Their viability was then determined by the Trypan blue dye-exclusion method.

### Antibodies

Cell surface staining was performed with phycoerythrin- or fluorescein isothiocyanate-labeled antibodies against the following proteins: CD11b, CD11c, CD16, CD32, CD45, FasL (Pharmingen, San Diego, CA); and TLR-1, -2, -3, and -4 (eBiosciences, San Diego, CA). For intracellular cytokine staining, we used phycoerythrin- or fluorescein isothiocyanate- labeled antibodies against TNF-α, IL-1β and IL-6 (R&D Systems, Minneapolis, MN). Appropriate isotype controls were used for every antibody.

### Cell surface and intracellular cytokine marker staining

Microglia were Fc blocked for 20 minutes using purified anti-CD16 antibody (Pharmingen). After washing, the cells were incubated with the fluorescent-labeled primary antibody or isotype control for 1 hour at 4°C. For the intracellular cytokine analysis, microglia were fixed with Cytofix/Cytoperm (BD Biosciences, San Jose, CA), washed in PermWash (BD Biosciences), and then stained with fluorescence-labeled monoclonal antibodies or isotype controls for 30 min at 4°C. Positive controls for all of the monoclonal antibodies consisted of A375 cells (E. G.). Approximately 1 × 10^4 ^live, gated events were assessed during fluorescence-activated cell sorting using an Epics XL-MCL cytometer (Beckman Coulter, Mountain View, CA) and analyzed using IsoContour software (Verity Software House, Topsham, ME).

### Phagocytosis assay

Fluorescent-labeled polystyrene microparticles (0.99 μm; Polysciences, 10 μL/tube) were coated with fetal bovine serum (FBS) diluted to 50% in phosphate-buffered saline and incubated with microglia (1 × 10^6^/tube) for 30 minutes at 37°C. The reaction was stopped by the addition of ice-cold phosphate-buffered saline. Cells were washed and analyzed by fluorescence microscopy (Nikon E400, Lewisville, TX) for internalized particles.

### Tumor cytotoxicity assay

1 to 2 × 10^6 ^U-87 MG (human malignant glioma) cells in 1 mL phosphate-buffered saline were labeled with 10 μL of 100× carboxy-fluorescein diacetate succinimidyl ester (CFSE, Cell Technology Inc., Minneapolis, MN) for 15 minutes at 24°C. The U-87 MG target cells were washed, resuspended in phosphate-buffered saline with 10% FBS, and incubated for 30 minutes at 37°C. Effector cells (microglia or PBMCs) were isolated and added at varying effector ratios to 1 × 10^5^target cells. Cultures were incubated for 4 hours or 24 hours, and 10 μL of 50 μg/mL propidium iodide was added to each culture before analysis by FACSCalibur Instrumentation and CellQuest Pro software (BD Biosciences, San Jose, CA).

### Statistics

The intracellular cytokine data was analyzed by Exact McNemars test for dichotomous categorical and matched data. The *in vitro *cytotoxicity data were analyzed using the Chi square test. The cutoff for statistical significance was *P *< 0.05.

## Results

### Purity of microglia isolated from human gliomas

**Figure 1:**
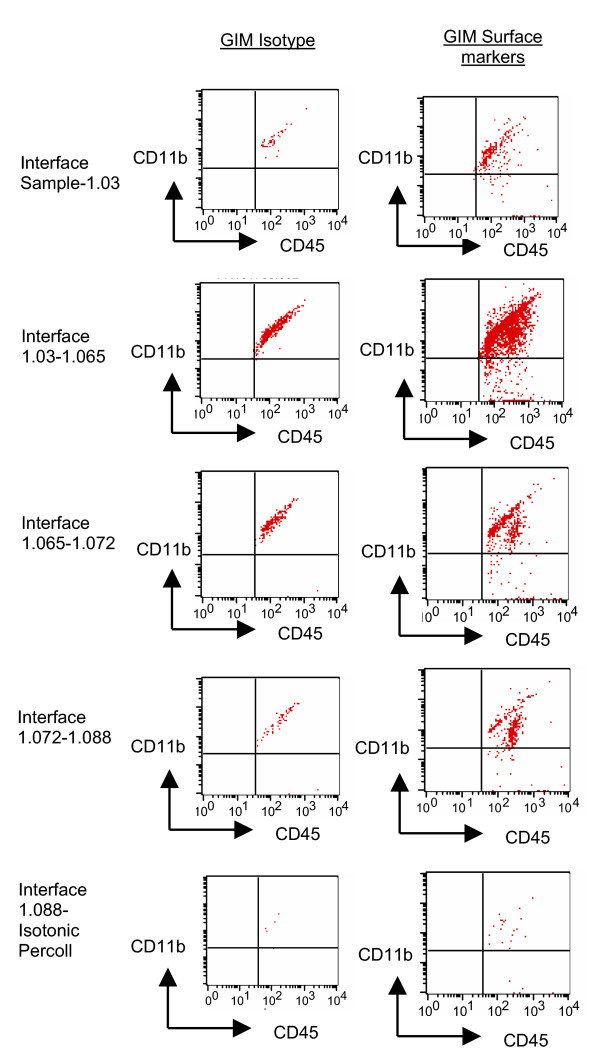
Isolation of GIM and expression of surface markers demonstrating the purity of the GIM population (CD11b vs. CD45) at each isolated fraction. Each interphase from the second percoll gradient was analyzed for expression of the surface markers CD11b and CD45. The gated cells in the upper right quadrant indicate the percentage of CD45^+ ^gated cells that are also positive for CD11b and represent GIM. An autofluorescent population was identified in both normal and GBM tissue samples during flow cytometry analysis and is also consistently present in the respective isotype controls. This population was excluded when calculating the percentages of positive fluorescing cells.

To determine the purity of our GIM isolation, cells were stained with fluorescent-labeled antibodies to CD11b, CD11c, and CD45, and the percentage of cells that were CD11b/c^+^CD45^low ^was determined using flow cytometry (Fig. 1). We can isolate a CD45^high ^population from peripheral blood, however, upon examining tumor homogenates before Ficoll purification we fail to see a CD11b/c+CD45^high ^seen in the previous murine studies. We observed the largest population of CD11b/c^+^CD45^+ ^cells at the interphase between the 1.03 and 1.065, unlike in the rodent studies where the interphase between the 1.065 and 1.072 gradients was identified to contain the largest fraction of CD11b/c^+^CD45^+ ^cells. The expression levels of CD45 and CD11b/c were not significantly different in the cells isolated from each interphase. Thus, in contrast to the rodent purification of microglia/macrophages in which distinct CD11b/c^+^CD45^high ^and CD11b/c^+^CD45^low ^cells were identified, we did not observe this heterogeneity in any of our GBM or normal brain tumor specimens. Our purified preparation was stained with antibodies to CD3, CD4, CD8, and CD56 and was negative for contaminating cell populations, including lymphocytes and NK cells (data not shown).

The average yield of microglia isolated from human GBM was 3.2 × 10^6 ^cells per gram of tumor (range: 1.8 × 10^5 ^- 3.7 × 10^7 ^cell/gram; n = 15) but was only 1.4 × 10^5 ^cells per gram of normal brain (range 4.6 × 10^4 ^- 3 × 10^5^; n = 5). This represented an average, although highly variable, 30% recovery and a 60 fold-enrichment of the microglia. The purity of GIM isolated from human glioma specimens can vary due to disparities in each excised individual tissue sample.

### TLR expression on GIM

**Figure 2:**
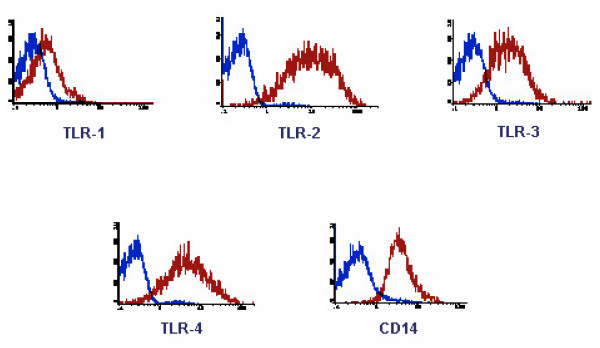
GIM express TLRs. GIM purity was determined as in Fig. 1 and the cells were double-stained for CD45 and TLRs. All CD45^+ ^expressing cells were gated and observed for TLR expression. In each histogram, the data plot on the left indicates the isotype control, and the second plot represents CD45^+ ^gated cells that expressed the respective TLR. These data are from one tumor specimen but are representative of at least five tumor tissue samples from human glioma patients.

To determine whether GIM expressed the TLRs, we stained our purified GIM with fluorescent-labeled antibodies to TLR-1, -2, -3, and -4. Although TLR-1 was not expressed in significant quantities, TLR-2, -3, and -4 were highly expressed on GIM (n = 5) (Fig. 2). The TLR expression profile was no different on microglia isolated from normal brain (n = 3) (data not shown).

### Cytokine expression by GIM

**Figure 3:**
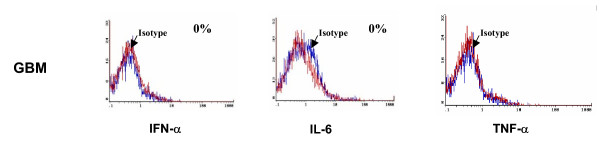
GIM do not secret innate cytokines. GIM purity was determined as in Fig. 1 and the cells were stained for intracellular cytokines IFN-α, IL-6, TNF-α. In each histogram, the data plot on the left indicates the isotype control, and the second plot represents respective cytokine producing cells. These data are from one tumor specimen but are representative of seven tumor tissue samples from human glioma patients and A375 control cells.

To determine if the microglia are activated within malignant gliomas (n = 7) and participating in innate responses, intracellular cytokine analysis was performed for IL-1β, IL-6, and TNF-α. GIM failed to produce any significant levels of these cytokines when compared to control cells (A375) and was no different from microglia isolated from normal brain tissue (n = 3) (Fig. 3, Table 1).

**Table 1:** Distribution of cytokine production by glioma infiltrating microglia from 7 representative human glioma patients. Positive intracellular staining was confirmed for all cytokines by either a reporter cell line (A375).

**Pathology**	**IL-1β **	**IL-6**	**TNF-α **
GBM	0%	0%	0%
GBM	0%	0%	0%
GBM	0%	0%	0%
GBM	0%	0%	0%
GBM	0%	0%	0%
AA	0%	0%	0%
AA	0%	0%	0%
Normal	0%	0%	0%
Normal	0%	0%	0%
Normal	0%	2%	0%
A375	58%	3%	5%

Percentages indicate CD11b^+^CD45^+ ^GIM gated on all CD45^+ ^cells and are adjusted to that of corresponding isotype controls.

Abbreviations: IL, interleukin; TNF, tumor necrosis factor; GBM, glioblastoma multiforme; AA, anaplastic astrocytoma.

### GIM are functionally capable of phagocytosis

**Figure 4:**
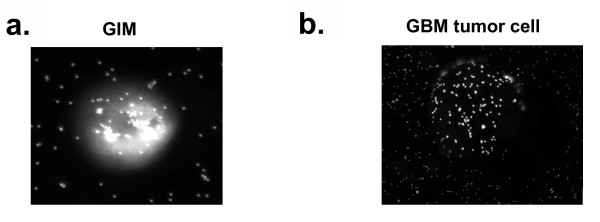
GIM can mediate phagocytosis. Cells were incubated with opsonized fluorescent beads and analyzed using fluorescence microscopy at 20× magnification. *A*, GIM containing phagocytosed beads. *B*, Control tumor cell that has fluorescent beads sticking to the surface but not internalized. Cells were analyzed through a z-stack to determine whether the beads were internalized or merely on the surface.

To determine whether GIM isolated from malignant gliomas were capable of mediating phagocytosis, GIM were incubated with opsonized fluorescent beads. GIM showed marked intracellular staining by the beads, indicating that phagocytosis was not impaired in GIM despite the immunosuppressive environment of gliomas (Fig. 4A). In contrast, control tumor cells bound the beads only on the surface of the cell (Fig. 4B).

### GIM can mediate non-MHC restricted anti-tumor cellular cytotoxicity

**Figure 5:**
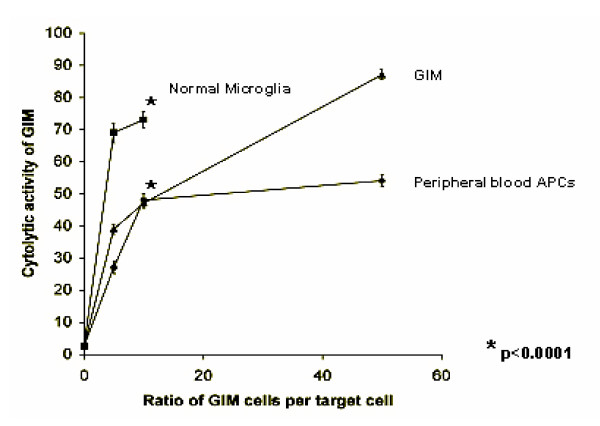
GIM can mediate non-MHC-restricted tumor cytotoxicity. GIM, peripheral blood mononuclear cells, and normal (5 × 10^6^, 1 × 10^6^, or 5 × 10^5^) microglia were each incubated as an effector population with 1 × 10^5 ^carboxy-fluorescein diacetate succinimidyl ester-labeled U-87 target cells, for an effector-to-target ratio of 50:1, 10:1, or 5:1, respectively. Propidium iodide was added after 24 hours incubation, and cells were analyzed by flow cytometry. Cells were first gated only on the carboxy-fluorescein diacetate succinimidyl ester^+ ^target population (excluding all other cells in the assay), and the propidium iodide expression on these cells was determined. The percentages of cytolytic activity of the effector cells were calculated (*Y *axis)(dead targets in upper right quadrant/[dead targets in upper right quadrant + live targets in lower right quadrant]) and plotted against each respective effector-to-target ratio (*X *axis). These data are from one tumor specimen and are representative of experiments from cells isolated from 3 different glioma patients. Bars at each data point represent 95% confidence intervals for each proportion. After 24 hours, GIM were functional in their tumor cytotoxic activity and comparable to PBMCs, however cytotoxic activity of GIM was significantly (*p < 0.0001) lower than that of normal microglia.

To ascertain whether GIM can mediate anti-tumor cytotoxicity, freshly isolated GIM were incubated as effectors with U-87 MG target cells (a cell line derived from malignant human glioma) at various effector-to-target ratios. As controls, microglia from normal brain tissue and PBMCs from the patient were incubated with U-87 MG cells in similar ratios. All effector cells were unstimulated before the assay and did not show significant cytotoxic activity after 4 hours (data not shown). After 24 hours, even at a 1:10 effector:target ratio, GIM were functional in their tumor cytotoxic activity (48.3% cells; 95% CI, 46.8-49.9) and comparable to PBMCs (46.9% cells; 95%CI, 45.5-48.2), however cytotoxic activity of GIM was significantly (p < 0.0001) lower than that of microglia isolated from normal brain tissue (72.4% cells; 95% CI, 69.7-75.0)(Fig. 5). We were not able to generate sufficient numbers of microglia from normal brain tissue to test higher effector:target ratios.

### Microglia express fas ligand at low levels

**Figure 6:**
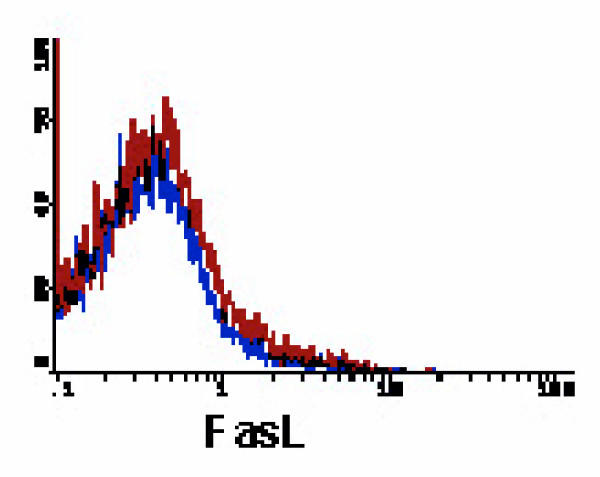
GIM cannot initiate Fas-mediated apoptosis. The histogram is depicted similar to that in Fig. 2. GIM were gated on CD45^+ ^expression and were analyzed for expression of FasL. These data are from one tumor specimen and are representative of at least 11 tumor tissue samples from human glioma patients.

To ascertain whether human GIM were preventing antitumor T cell activity via Fas-FasL-mediated apoptosis of T cells, the GIM were stained with an anti-FasL antibody. In contrast to the murine findings, human GIM did not express FasL or expressed it at very low levels (n = 11)(Fig. 6), indicating that Fas-FasL-mediated apoptosis is not a predominant mechanism of immune evasion by GIM in humans.

## Discussion and conclusion

This study evaluates for the first time the innate immunologic phenotype and function of human microglia isolated from brain tumors. Activation of innate immunity triggers the subsequent activation of adaptive immune response [24,25] that are essential for tumor eradication/suppression. An ongoing debate is whether the immune system can recognize the presence of a tumor. Although it has been argued that tumors fail to provide sufficient pro-inflammatory responses, others have recently suggested otherwise [26,27]. Mechanisms that induce innate immune responses similar to microbial pathogens include dying cells producing uric acid [28]; heat shock proteins [29-31]; and extracellular matrix derivatives, such as hyaluronic acid [32] or heparin sulfates [33], which are all TLR-4 agonists. All these materials are prodigiously produced by gliomas [34-37] indicating that innate immunity can be potentially activated by these glioma factors. Our data shows that similar to the murine system [16], there is TLR expression on the GIM. The interaction of the microglia with TLR agonists should lead to the induction of a plethora of inflammatory mediators, such as TNF-α, IL-1 and IL-6. These cytokines subsequently induce the local inflammatory response. However, none of these cytokines were produced by the microglia within the immunosuppressive microenvironment of the malignant glioma. It is important to note that, unlike the phagocytosis of pathogens that is followed by the induction of inflammatory mediators, phagocytosis of apoptotic and senescent cells is immunologically silent and does not lead to the induction of inflammatory responses. Thus, despite the elaboration of factors such as heat shock proteins and hyaluronic acid that could activate innate immunity on microglia, the "balance" of micro-environmental influences including apoptotic glioma cells and immunosuppressive cytokines impede innate immune activation as reflected by cytokine production.

GIM can participate in the initial steps of innate immunity since they are capable of phagocytosis. However, due to the limitations in obtaining normal brain tissue, we cannot discount the possibility that this phagocytic ability of GIM might be impaired when compared to that of microglia isolated from normal brain. Indeed we find that GIM, while capable of non-MHC mediated cytotoxicity, they are significantly less able to participate in tumor cytotoxic activity compared to normal microglia. The direct cytotoxicity of GIM against tumor cells is tempered by the fact that immunosuppressive cytokines/factors that influence the function of GIM are withdrawn for 24 hours. However, the U-87 glioma cell line secretes the immunosuppressive cytokine TGF-β, in part recapitulating the *in vivo *scenario. *In vivo *cytokines such as IL-10 prevent macrophages from becoming tumoricidal by inhibiting the production of TNF and nitric oxide. Because the assay requires 24 hours, the effects of the tumor microenvironment are in part withdrawn while the assay is in progress. Thus, the relative ability of GIM compared to normal microglia to mediate cytotoxicity may be even more profound *in vivo*.

The role of FasL within innate immunologic responses is not straightforward. For example, when tumors express soluble FasL, immunologic responses appear to be suppressed. However, when FasL is expressed in a membrane-bound form, neutrophil-mediated inflammation is induced, and both the innate and adaptive immune systems are activated [17]. Contradictory results have demonstrated that high levels of expression of FasL can inactivate neutrophils [19]. Murine microglia have previously been reported as expressing FasL [20]. We wanted to determine whether FasL was similarly a potential immunotherapy target in human GIM; however, we did not find any significant expression of FasL on microglia across a wide array of grades of gliomas and metastatic cancers. Therefore, modulating techniques directed at FasL on human GIM are not likely to be successful. However, this does not rule out a potential immunological benefit of modulating FasL on the tumor cells.

This study is the first to our knowledge to characterize immune activation at the level of the innate arm on microglia in glioma patients. Of course, we cannot exclude the possibilities that activation of the innate immune system could occur on another CNS cells beside microglia or that innate immune activation could occur via draining TLR agonists to the cervical lymph nodes. The activation of innate immunity on GIM through select TLRs may be a future approach to enhancing the incorporation and activation of adaptive immune responses, but a careful screening through the panel of agonists will be necessary because not all TLR agonists may cause activation, especially in the presence of immunosuppressive cytokines/factors. While it is important to further characterize these innate functions and understand the role they play in anti-tumor immune responses, our data appears to demonstrate that some of the innate immune functions remain unaffected despite the immunosuppressive glioma environment and can perhaps be manipulated to develop effective immunotherapeutic responses to gliomas.

## Competing interests

The author(s) declare that they have no competing interests.

## Authors' contributions

SFH participated in the design of the study, carried out the flow cytometry, phagocytosis and cytotoxicity assays and helped draft the manuscript, DY carried out the microglia purification from tumor specimens and provided technical assistance for the assays, DS carried out the statistical analyses, EG provided input in the design of the study and ABH conceived of the study, participated in the design and coordination and helped draft the manuscript.
